# Whole Genome Sequencing in the Management of Non-Tuberculous Mycobacterial Infections

**DOI:** 10.3390/microorganisms9112237

**Published:** 2021-10-27

**Authors:** Matúš Dohál, Igor Porvazník, Ivan Solovič, Juraj Mokrý

**Affiliations:** 1 Biomedical Center Martin, Department of Pharmacology, Jessenius Faculty of Medicine, Comenius University, 036 01 Martin, Slovakia; juraj.mokry@uniba.sk; 2 National Institute of Tuberculosis, Lung Diseases and Thoracic Surgery, 059 81 Vyšné Hágy, Slovakia; igor.porvaznik@vhagy.sk (I.P.); solovic@hagy.sk (I.S.); 3 Faculty of Health, Catholic University, 034 01 Ružomberok, Slovakia

**Keywords:** whole-genome sequencing, non-tuberculous mycobacteria, diagnostics

## Abstract

Infections caused by non-tuberculous mycobacteria (NTM) have been a public health problem in recent decades and contribute significantly to the clinical and economic burden globally. The diagnosis of infections is difficult and time-consuming and, in addition, the conventional diagnostics tests do not have sufficient discrimination power in species identification due to cross-reactions and not fully specific probes. However, technological advances have been made and the whole genome sequencing (WGS) method has been shown to be an essential part of routine diagnostics in clinical mycobacteriology laboratories. The use of this technology has contributed to the characterization of new species of mycobacteria, as well as the identification of gene mutations encoding resistance and virulence factors. Sequencing data also allowed to track global outbreaks of nosocomial NTM infections caused by *M. abscessus* complex and *M. chimaera*. To highlight the utility of WGS, we summarize recent scientific studies on WGS as a tool suitable for the management of NTM-induced infections in clinical practice.

## 1. Introduction

Non-tuberculous mycobacteria (NTM) are all species of bacteria within the genus *Mycobacterium* (*M*.), except *M. tuberculosis* and *M. leprae*; so far, more than 180 species have been identified [1]. NTM are environmental, opportunistic pathogenic bacteria and only a limited number of species cause infections that primarily affect the lungs (pulmonary disease), skin, soft tissues, lymph nodes, but are also responsible for surgical wound infections, implant-associated and catheter infections [2,3,4]. The pulmonary form of the disease may be manifested by hypersensitivity pneumonitis, nodular bronchiectasis, or fibrovascular disease. Infections caused by NTM have risen rapidly in recent decades, and in many developed countries, the number of cases has exceeded the incidence of pulmonary tuberculosis [5]. In addition, some species of mycobacteria are also associated with increased mortality in domestic, wild animals, or aquatic organisms, thus contributing to high economic damage and negative environmental impact [6,7,8].

Species belonging to the *M. avium* complex (*M. chimaera*, *M. intracellulare*, *M. avium*) are the most widespread globally, but species within *M. abscessus* complex (*M. abscessus* subsp. *abscessus*, *M. abscessus* subsp. *boletti*, *M. abscessus* subsp. *massiliense*) also pose a great threat due to high degree of resistance [9,10]. The increasing incidence of infections caused by NTM is associated with demographic changes, aging population, and an increasing number of patients with chronic diseases (e.g., chronic obstructive pulmonary disease, cystic fibrosis (CF), cancer, diabetes), including immunosuppressive conditions [11]. However, the disease can also occur in patients without obvious predisposition, which may be related to specific gene mutations encoding changes in the function of the immune system [12].

The prolonged treatment (at least 18 months) regimens of NTM infections based on the administration of the combination of several antibiotics are often associated with poor patient outcomes and cure rates ranging from 30–50% for *M. abscessus* infections to 80–90% for *M. malmoense* infections [13]. The lipid-rich hydrophobic cell walls of NTM are optimal for biofilm formation. These structures are effectively resistant to antimicrobial agents and allow bacterial colonies to persist on living and non-living surfaces. In addition, treatment regimens and management of clinical symptoms vary across the species of NTM, so proper identification of the causative agent is important in clinical practice [14,15].

In diagnostics of NTM, 3 or more sputum specimens from a single patient are necessary. However, due to the high risk of contamination from the microflora of the oral cavity, the diagnosis can be made from a single bronchoscopic sample or a lung biopsy [16]. In the past, NTMs have been characterized by their growth rate (slow-growers: forming visible colonies in seven or more days; rapid-growers: forming visible colonies in less than seven days) and the type of pigment produced. Rapid-growing NTM (the most clinically relevant species are *M. chelonae*, *M. fortuitum*, *M. abscessus*) do not produce pigment, whereas slow-growing NTM (the most clinically relevant species are M. avium complex, *M. kansasii*, *M. marinum*, *M. simiae*, *M. haemophilum*, *M. xenopi*) are divided into 3 groups according to the type of pigment produced: nonchromogens, photochromogens, and scotochromogens. The slow-growing NTM are more prevalent and present a higher risk of drug resistance than fast-growing ones [17]. In general, smear microscopy is the diagnostic method preferred in most clinical laboratories in countries worldwide, due to its low cost. However, the sensitivity of smear microscopy is only 60–70%, and the inability to diagnose extrapulmonary forms of mycobacterial diseases significantly reduces its usefulness [18]. Despite these facts, smear microscopy and culture are still gold standards in diagnostics of mycobacteria. Traditional biochemical and immunological assays or high-performance liquid chromatography used to identify NTM are time-consuming, insensitive, therefore have been replaced by molecular methods such as line probe hybridization, polymerase chain reaction (PCR), real-time PCR, and DNA sequencing. Limitations of conventionally used 16s rRNA gene sequencing (the approved standard for identification, classification, and quantitation of microbes) include the inability to distinguish *M. abscessus* subspecies, as well as misidentification of *M. chimaera* as *M. intracellulare*, therefore other conserved genes are used (e.g., *rpoB*, *hsp*6 and 16S–23S internal transcribed spacer). Some commercial kits, based on PCR, are available, such as AccuProbe (Hologic Inc., Marlborough, USA), INNO-LiPA Mycobacteria (Fujirebio Europe, Ghent, Belgium), Speed-oligo Mycobacteria (Vircell, Granada, Spain), and GenoType NTM-DR (Hain Lifescience, Nehren, Germany), with a narrow spectrum of detected NTM species and limited sensitivity and specificity [19].

The whole-genome sequencing (WGS) method allows clinicians to overcome the limitations of the conventional tests used for the diagnosis of mycobacterial infections and better understand the global diversity of NTM species [20]. WGS can identify all single nucleotide polymorphisms (SNPs) associated with resistance, as well as phylogenetic SNPs characteristic for individual NTM species [21]. In addition, WGS also contributes to the diagnosis of mixed NTM infections associated with two or more particular species [22]. Sequencing data should be also used in molecular epidemiology analysis as they provide a detailed insight into transmission dynamics of NTM (e.g., identification of hospital outbreaks) [23]. Although WGS technology is currently not available in all clinical settings, however, the purchase price is gradually reduced, and in some reference mycobacteriological laboratories this method is implemented into routine practice for identification of mycobacteria and genotypic drug susceptibility testing of *M. tuberculosis* [24].

In this mini-review, we summarize the latest scientific publications focused on WGS as a suitable tool for the management of NTM infections.

## 2. WGS Perspectives for Diagnostics and Characterization of Resistance Patterns of NTM

The early symptoms of NTM disease are usually non-specific. Patients complain of chest pain, productive cough, shortness of breath, fatigue, and fever [25]. In clinical laboratories in countries with a high incidence of tuberculosis, the diagnosis of mycobacterial infections is based mainly on the culture detection of acid-fast bacilli in a sputum sample and chest radiograph (X-ray or high-resolution computed tomography findings), which complicates the differentiation of NTM from *M. tuberculosis* complex due to morphological similarities together with identical clinical symptoms, and can lead to incorrect and ineffective treatment regimen [26]. In this context, Advani et al. performed a WGS study on *M. tuberculosis* isolates and found that almost 20% of all isolates contained NTM mycobacteria, indicating coinfection [27]. Ongoing NTM infection symptoms are often non-specific and can be also misdiagnosed as chronic obstructive pulmonary disease, chest infection of unknown cause, or, more recently, COVID-19. Therefore, the correct species-level identification of pathogens is essential in clinical practice.

**Figure 1:**
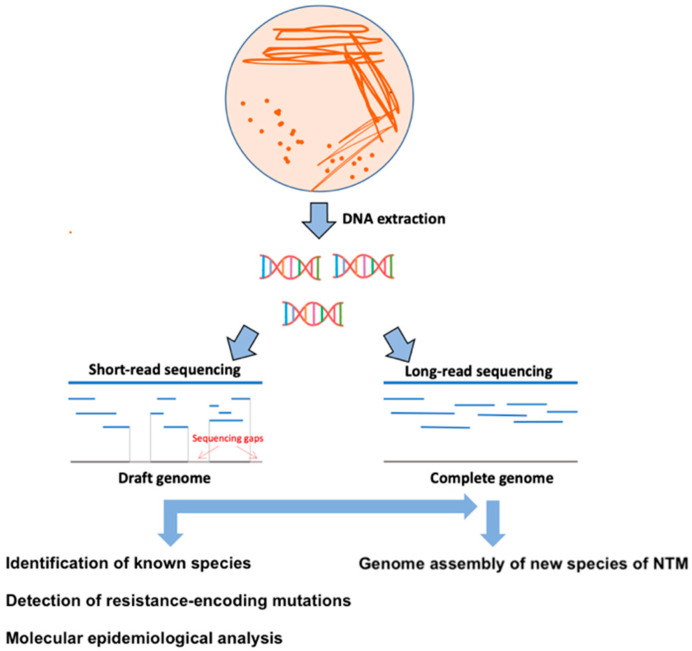
Schematic representation of the WGS methodological procedure. In the first step, DNA is extracted from colonies grown on a solid or liquid culture medium. Subsequently, sequencing libraries are prepared for short- or long-read sequencing. In the last step, the sequencing data are bioinformatically processed for genotyping, identification of resistance patterns, molecular-epidemiology analysis, and assembling of complete genomes of new NTM species.

WGS technologies provide the possibility of taxonomic reconstruction and identification of unique families of genes occurring within the genus *Mycobacteria* with biomedical application in the development of new reliable diagnostic tools [1]. These data are also useful in identifying rare NTM, the clinical significance of which is greatly underestimated, because they remain unrecognized in diagnostic laboratories, highlighting the utility of WGS compared to traditional diagnostic methods [28]. In general, NTM colonies grown on solid media (e.g., Lowenstein-Jensen) or in liquid media (Middlebrook 7H9 broth) of automated incubation system (BACTEC MGIT 960 system, Becton, Dickinson and Company, Franklin Lakes, NJ, USA; VersaTREK system, Thermo Fisher Scientific Waltham, MA, USA; MB/BacT Alert 3D, bioMérieux, Marcy-l’Étoile, France) proceeded from clinical (pulmonary-sputum; extrapulmonary-blood, tissues) or environmental samples (microbiological swabs) are the most commonly used materials for diagnostic and sequencing purposes (Figure 1).

Quan et al. tested the efficacy of WGS for NTM classification compared to conventional available line probe assays (GenoType CM test, GenoType Mycobacterium MTBC or GenoType Mycobacterium AS test). Their results confirmed the high agreement of the individual methods, of which sequencing showed the highest discriminatory power, including within complex diversity (important especially within *M. avium* complex and *M. abscessus* complex) and identification of rare species, for example, *M. ratisbonense* or *M. tomidae* [29]. This is due to the limited number of amplified genes included in the conventional assays in combination with higher homology between mycobacterial species in contrast with other bacterial species [30]. The disadvantage of WGS compared to line probe assay is a slightly longer turnaround time (3–15 days). Similarly, the study by Yoon et al. confirmed 100% compliance of WGS and classical molecular genetic methods (PCR and direct sequencing) in the diagnostics of *M. avium* subsp. *hominissuis*, *M. intracellulare*, and *M. abscessus* [21].

In addition, WGS has proven to be a suitable tool to distinguish between relapse and reinfection of NTM caused predominantly by *M. ulcerans* or *M. abscessus* [31,32]. WGS also allows the identification of NTM at the clone level, thus allowing the form (relapse/reinfection) of the ongoing disease to be determined [33]. Flohr et al. have applied WGS in diagnostics of factitious disorders caused by NTM that may be misdiagnosed as a recurrent or chronic forms of infection [34]. The need to correctly clarify the form of the disease is highlighted by the high recurrence rate of some NTM infections, which is up to 10–48% after successful treatment [35,36].

Accurate diagnosis of NTM is crucial in patients with structural or inflammatory lung disease such as CF, non-cystic fibrosis bronchiectasis, or chronic obstructive pulmonary disease, as these emerging pathogens cause an accelerated decline in lung function and do not respond to aggressive antibiotic treatment in up to 50% of cases [37]. The most important predisposing factors in CF patients include nutritional condition, presence of bronchiectasis, and impaired mucociliary clearance [38]. In addition, the prevalence of NTM in CF patients is almost 10,000-fold higher compared to the rest of the population [39]. The two most common NTM species isolated in CF belonging to *M. avium* complex (representing 75% of NTM infections in CF) and *M. abscessus* complex (most common *M. abscessus* subsp. *abscessus* and *M. abscessus* subsp. *massiliense*) with prevalence ranges from 3 to 23% in CF centers worldwide [40]. The WGS of *M. abscessus* ATCC19977 showed that this species encoded virulence factors typical of both mycobacterial pathogens and common CF pathogens (*Pseudomonas aeruginosa* and *Burkholderia cepacia*) [41]. Trovato et al. confirmed that WGS can more effectively discriminate *M. abscessus* subspecies in patients with CF in comparison with a variable number of tandem repeat (VNTR) methods [42]. Similar results were shown in a recent study by Redondo et al. in which they successfully diagnosed individual subspecies of *M. abscessus* in CF- and non-CF patients [43]. Furthermore, *M. abscessus* complex infections are seen as a contraindication to lung transplant in some CF patients (currently the only cure for CF), due to the high likelihood of re-infection [44]. The risk of disseminated post-transplant infection is potentiated in patients with a positive presence of *M. abscessus* in sputum before lung transplantation [45,46]. Kavaliunaite et al. confirmed the association between a particular *M. abscessus* sequence type (ST) and the success of heart and lung transplantation in CF patients [47]. These results are particularly important as reported cases are increasing, and in some studies, the rate of NTM infections in lung transplants was up to 8% and was directly related to an increased risk of death [48,49]. In contrast, post-transplant infections caused by *M. avium* complex are less frequent and associated with a higher cure rate [46]. To decrease the risk of mortality, clinicians should consider the pretransplant or posttransplant (colonized by rapidly-growing NTM) chemoprophylaxis in all patients undergoing the lung transplant [50].

The benefit of WGS has also been shown in the management of multiple-NTM infections, the diagnosis of which is necessary for the correct setting of the treatment regimen and outcome expectations, as well as a better understanding of species diversity in various anatomical regions of the lung [51]. A recent WGS study by Shaw et al. analyzing the genomes of *M. abscessus* isolates revealed the multiple subpopulations diversity with different antimicrobial resistance profiles in children with CF. The results of this study are also important for the clinical diagnosis of the disease, as they confirmed a higher diversity of *M. abscessus* in samples of lungs, chest wounds, and pleural fluid compared to sputum [52]. Sequencing data provided a more detailed subspecies classification in mixed cultures compared to conventionally used methods [53,54]. WGS also contributed to the finding of a high interspecies and intraspecies *M. avium* complex diversity in a patient with an ongoing pulmonary form of the disease. These results elevate the clinical potential of WGS, as commercial probes resolve *M. avium* species only to the complex level [55]. Greninger et al. also used WGS technology to diagnose polymicrobial infection in brain abscess caused by rare, rapidly growing *M. immunogenum* and *M. llatzerense* [56].

For diagnostic purposes, better treatment results, and expanding our knowledge of mycobacterial diseases, it is necessary to identify and classify new species/subspecies of NTM [28,57,58]. Describing the complete genomes of the novel NTM requires a combination of short-read (e.g., Illumina MiSeq, San Diego, CA, USA) and long-read (e.g., MinION, Oxford, UK; PacBio SMRT, Pacific Biosciences, CA, USA) sequencing technologies (Figure 1 and Table 1) [59]. Short-read sequencing is characterized by high accuracy but does not provide information about the complete genome (including G + C rich regions, recombination, repetitive PE/PPE regions, deletions, and insertions) [60]. In contrast, long-read sequencing technologies have increased read lengths 100- to 1000-fold with slightly lower accuracy compared to NGS platforms and can span much larger repeat regions than NGS, thus contributing to new genome assembly [61].

Matsumoto et al. identified 27 genomes belonging to the novel NTM. In addition, sequencing data obtained using long-read sequencing technology were used in the same study to develop a multi-locus sequence typing database and software to identify mycobacteria. Sensitivity and specificity were highest compared to conventional methods in the characterization of 29 species of NTM [62]. Hendrix et al. combined Illumina and MinION sequencing for whole-genome assembling of *M. kubicae* isolates. Their results have provided detailed information regarding the resistance, virulence and persistence encoded on the chromosome or plasmids of these rare, clinically important pulmonary disease-causing NTM [63]. A recent study showed that MinION sequencing technology can be used to detect mycobacteria directly from clinical samples and diagnose the disease within 20 min, therefore setting the treatment regimen in a clinically relevant time frame [64].

Although NTM resistance is becoming a growing threat, susceptibility testing in clinical laboratories is performed primarily by broth microdilution and macrodilution phenotypic methods which may be difficult to evaluate [65]. Additionally, the treatment for NTM infections usually includes a combination of macrolide antibiotics (such as azithromycin and clarithromycin) with aminoglycosides (for fast-growing NTM) or first-line antituberculosis drugs (for slow-growing NTM). However, for some combinations, clinical breakpoints are not well defined, therefore, the effectiveness of treatment can be greatly reduced [5,66]. Generally, many NTM species are naturally resistant to specific drugs, however, improperly adjusted and prolonged treatment regimen leads to the development of acquired resistance in originally drug-sensitive bacterial strains [67]. A key factor that plays a role in natural NTM resistance is the low permeability of the mycobacterial cell wall [68]. However, *M. abscessus* exhibits intrinsic high-level resistance to ethambutol and fluoroquinolones. This resistance is predominantly associated with mutations in the *embB* (encoded resistance to ethambutol) and *gyrA* (encoded resistance to fluoroquinolones) genes [69]. *M. abscessus* and *M. avium* exhibit intrinsic resistance to rifampicin, primarily due to mutations in the *rpoB* gene and the presence of the MAB_0591 gene [65]. Another important mechanism of intrinsic resistance is the overexpression of efflux pumps, which is responsible for the resistance of *M. abscessus*, *M. avium*, and *M. intracellulare* to bedachilin and clafazimine [70,71,72]. Acquired drug resistance in mycobacteria is often mediated by a chromosomal mutation in genes encoding targets [73]. With the utility of WGS, it is possible to describe the underlying mechanisms behind the drug resistance of NTM species and increase the effectiveness of treatment regimens [74]. Wetzstein et al. studied the efficacy of WGS in predicting *M. abscessus* resistance to macrolides and aminoglycosides. The results obtained by WGS were shown to be in full agreement with the Genotype NTM-DR line probe assay and phenotypic drug susceptibility testing [75]. Lipworth et al. used sequencing data obtained from *M. abscessus* species for the identification of new mutations in erm and rrs genes potentially associated with macrolide antibiotic resistance. These mutations are not currently included in traditional genotyping tests (such as GenoType NTM-DR; Hain Lifescience, Nehren, Germany) which may therefore show false-negative results [76]. Similarly, Chen et al. performed WGS on clofazimine-resistant strains of *M. abscessus*. Their results revealed several high-confidence gene mutations (in MAB_2299c, MAB_1483, and MAB_0540 genes) involved in resistance to this drug, whose use is increasingly preferred due to limited treatment options [77]. In addition, *M. abscessus* subsp. *abscessus* possesses the erm gene, while *M. abscessus* subsp. *massiliense* harbor the nonfunctional variant of this gene that lacks the inducible resistance phenotype, so it is important in clinical practice to distinguish between these two subspecies [62]. In contrast, a recent WGS study involving a global collection of *M. abscessus* complex isolates revealed a 10% higher frequency of mutations in the rrl gene encoding macrolide resistance in *M. massiliense* compared to *M. abscessus* subsp. *abscessus* [78]. Based on WGS-data, Yoshida et al. developed special DNA-chromatographic and PCR-based diagnostic assays for discriminating macrolide-resistant/susceptible subspecies of *M. abscessus* (*M. massiliense*, *M. abscessus*, *M. bolletii*). The agreement rate with WGS was 99.7% [79]. Realegano et al. developed a novel clinical whole genome sequencing assay to determine the resistance of *M. abscessus* to clarithromycin and amikacin. The accuracy of resistance prediction compared to phenotypic results was 100% for both drugs [80].

However, the increased incidence of resistant strains is leading to the development of new treatment strategies such as treatment with lytic phages. Dedrick et al. used WGS for a better understanding of phage resistance mechanisms of *M. abscessus* [81].

## 3. WGS Perspectives in Tracing the Transmission of Nosocomial NTM Infection

Nosocomial NTM infections have been defined as infections directly associated with a hospital stay, or their symptoms may appear immediately after discharge [82]. The first cases of nosocomial NTM infections caused by *M. fortuitum* have been recorded since the beginning of the 20th century [83]. Nowadays, NTMs are classified as well-adopted nosocomial pathogens with a wide range of clinical manifestations and hospital sources of infection [26]. Their adaptation to the hospital environment results from their resistance to disinfectants and sterilants, low nutrient requirements, and ability to form a biofilm [2]. These nosocomial infections are usually extrapulmonary: bloodstream infections, postoperative wound infections, implant-associated infections, spinal infections, etc. [84].

**Figure 2:**
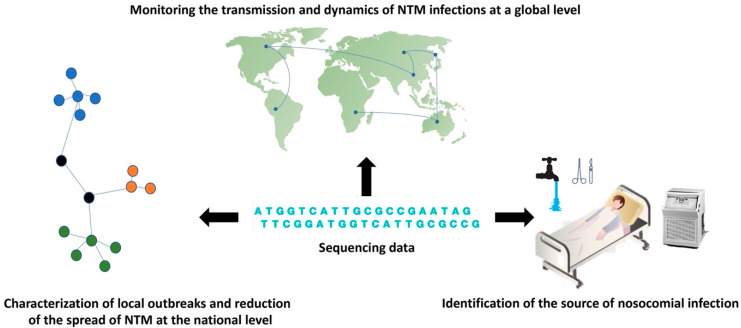
The contribution of WGS in the molecular epidemiology of NTM infections. Sequencing data can be used to describe the global spread and dynamics of NTM infections, to characterize local outbreaks, and also to identify sources of infection in hospital settings.

Genotyping of individual NTM strains is crucial for tracing the nosocomial outbreaks and epidemiological investigation. Previously preferred typing methods, including biotyping, serotyping, and antibiograms, are no longer performed in reference laboratories due to their insufficient discriminatory power. Current genotyping techniques based on 16S rRNA sequencing, pulsed-field gel electrophoresis, VNTR, and spoligotyping or plasmid typing, are relatively easy to perform in a short time frame and require only equipment available in most laboratories [85]. However, these techniques show some limitations in recognizing similarities and differences between close phylogenetically similar strains, which are reflected in the misalignment of these strains within clusters and by the inability to identify their common ancestor [86,87]. Recent studies confirmed that WGS exhibits higher discriminatory power and a wide range of uses in molecular-epidemiology analysis than current genotyping techniques (Figure 2) [88,89]. The results of Harris et al. confirmed higher resolution of WGS in comparison with VNTR profiling in distinguishing NTM subspecies into individual clades [90]. The WGS phylogenomic analysis is usually based on the identification of a massive number of different single nucleotide variants between isolates to cluster and compare genomes, infer relatedness and identify the source of infection. A factor complicating the diagnosis of the disease as well as the tracing for the source of the infection is the long incubation period, which can last several months or years [91].

In the past, it was believed that NTM were transmitted to humans only after exposure to the environment, including soil particles or water droplets containing mycobacteria. The ability of NTM to survive and proliferate in this environment is provided by a lipid-rich and triple-layered outer membrane that protects the cells against acids, high temperatures, and ultraviolet light [2]. However, WGS served to shift this paradigm by allowing the characterization of patient-to-patient transmission events and contributing to an increase in reports of outbreaks of nosocomial NTM infection. In 2012, Aitken et al. reported person-to-person transmission of *M. abscessus* subsp. *massiliense* among the patients with CF for the first time [92]. Further studies followed documenting healthcare-associated cross-transmission of *M. abscessus* between CF patients, including pediatric patients in national CF centers (with less than 7 SNP difference between isolates) [25,93,94,95,96]. In many of these studies, the specific lineage was isolated only from patients and not from environmental sources. Moreover, Bryant et al. performed a WGS-based study of a collection of *M. abscessus* isolates obtained from worldwide (Europe, Australia, the Republic of Ireland, and the United States) community of CF patients (1080 isolates from 510 CF patients). The results confirmed the global circulation of three dominant, genetically similar, multi-drug resistant *M. abscessus* clones (74% isolates were clustered) that are transmitted within CF clinics [97,98]. These clones are associated with poorer clinical outcomes and showed increased intracellular survival, pathogenicity, and virulence in macrophage and mouse models [99]. Interestingly, the study of Lipworth et al. showed that most genetically related *M. abscessus* clones isolated from CF patients had no identifiable relevant epidemiological traceback [100]. In addition, Bronson et al. performed a comparative genomic analysis of 1279 *M. abscessus* complex genomes from CF and non-CF patients. The results of the study identified small pairwise SNP distances and similar phylogenetic patterns between isolates from patients with CF and without CF, suggesting that these cases originate from a recent common ancestor [77]. However, how these clones are widespread worldwide, either by asymptomatic transmission or by the environment, has not yet been confirmed. Nevertheless, the prevention and control guidelines recommend isolating infected patients to prevent further transmission of *M. abscessus* within healthcare facilities [101].

In addition to *M. abscessus*, the transmission of *M. avium* complex (predominantly *M. chimaera*) was also studied. A recent study defining the genetic relationship of *M. avium* complex isolates between patients and between patients and their environment confirmed the minimal likelihood of patient-to-patient transmission and identified the hospital water distribution system as the main reservoir of NTM [21]. Moreover, WGS has contributed to the characterization of the global outbreak of nosocomial infections caused by *M. chimaera* in patients who underwent cardiac surgery. These infections are usually presented as prosthetic valve endocarditis, disseminated infections, or infections of vascular grafts [102]. The first outbreak investigation began in 2013 at the University Hospital Zurich in patients with confirmed presence of *M. chimaera* after cardiac surgery [103]. WGS epidemiological analysis in other studies demonstrated that aerosols from the water tanks in the heater-cooler units contaminated with *M. chimaera* can be airborne disseminated to patients and the surrounding environment [87,104,105,106,107,108]. In this context, Götting et al. found the genetically related *M. chimaera* contaminated the air of the heater-cooler units as well as medical instruments in the operating room [109]. Most of the reported nosocomial outbreaks included patients who were exposed to heater-cooler units from the same manufacturer during the open-heart surgery (LivaNova, London, United Kingdom). A subsequent study confirmed the culture positivity of *M. chimaera* in water samples on the manufacturer’s site and thus confirmed the source of infection [107]. Despite the aggressive therapy with the combination of at least 3 antibiotics (usually including the ethambutol, rifamycin, and macrolide) and surgical removal of any involved devices, 50% of the patients died from complications associated with *M. chimaera* infection. This is due to the ability of *M. chimaera* to replicate in the tissues and disseminate to other organs even with previous long antibiotic therapy [110]. The Centers for Disease Control and Prevention (CDC, Atlanta, GA, USA) has also recently released guidelines for the management and diagnostics of heater-cooler unit-associated NTM infections, in which WGS plays a crucial role [111]. The available data also suggest that the risk of *M. chimaera* infection in patients after cardiac surgery is almost identical to the level of risk of infection in HIV-positive patients [112]. Moreover, clinical symptoms and laboratory findings are often nonspecific, therefore, every patient with a diagnosis of sarcoidosis or culture-negative endocarditis after exposure to a heater-cooler unit should be considered as *M. chimaera* positive [113]. Surgical site infection by *M. chimaera* may also occur, predominantly in patients after plastic surgery. WGS showed the genetic relatedness of isolates from patients and the samples of tap water at the surgical clinic, therefore exposure of the wound to any non-sterile water should be totally forbidden before complete wound healing [114]. Insufficient sterilization of surgical instruments and water has been suggested as a source of *M. chimaera* in many other studies, highlighting the utility of WGS in clinical practice [115,116].

Labuda et al. used the WGS approach for characterization of a new species of rapidly growing NTM (*Mycobacterium* FVL 201832) and identification of saline flushes as a source of nosocomial bloodstream infections in oncology patients. These results contributed to the consideration of new state regulations to monitor and reduce the spread of NTM in oncology clinics [117]. Adherence to these regulations is essential, as recent studies have confirmed that *M. avium* complex infection increases tumor-genes inflammatory responses which could lead to the development of lung and breast cancer and other complications in oncological patients [118]. Similarly, a study by Inkster et al. revealed hospital water supply contaminated with *M. chelonae* as a potential source of infection in hemato-oncology patients [119]. The genetic diversity of unique species of *M. shigaense* (belonging to *M. simiae* complex) causing cutaneous infections have been studied using WGS technology [120].

## 4. Conclusions

NTM infections are associated with a substantially impaired quality of life, increased morbidity, and mortality, and high treatment costs, therefore, it is essential to use sufficiently sensitive diagnostic methods such as WGS. Even though the cost of WGS samples decreases every year, the implementation of this method in clinical diagnostic laboratories requires a higher initial capital and well-trained personal therefore is not available in the mycobacteriology laboratory in resource-limited countries. It is also time-consuming compared to traditionally used tools, as culture cultivation must be performed prior to DNA isolation. Therefore, more research is focused on WGS directly from clinical material. Another limitation in the implementation of WGS into routine practice is also caused by the bioinformatics processing of sequencing data. Nowadays, it requires demanding software and programming skills. However, technological advances in the coming years will lead to the development of easy-to-use online webtools such as TB-Profiler (https://tbdr.lshtm.ac.uk/, accessed on 22 October 2021) or PhyResSE (https://bioinf.fz-borstel.de/mchips/phyresse/, accessed on 22 October 2021), which are used to diagnose and determine the complete resistance profile of *M. tuberculosis*.

In contrast, the WGS-data are more comprehensive as these allow the precise identification of novel NTM species from human samples, prediction of genes for virulence, intracellular existence, disease, defense, and toxic compounds of NTM, thus emphasizing other applications of this method (Table 2) [58,121,122]. Moreover, extended datasets of clinical phenotypes and bacterial DNA sequences could resolve ambiguities in the pathogenesis of NTM. We also assume that in the future, the results of WGS will lead to wider use of personalized medicine and thus increase the effectiveness of treatment regimens.

In conclusion, the more NTM genomes are sequenced, the more they will contribute to the global tracing of NTM infections and the description of important genetic determinants, thus the development of novel diagnostic tools and new therapeutic targets.

## Figures and Tables

**Table 1:** Comparison of basic sequencing platforms used for complete genome assemblies of new NTM species.

Sequencing Platform	Read-Length (bp)	Output	Accuracy	Advantage
Illumina MiSeq	25–300	>50 Gb	>99%	Higher read quality A lower amount of input DNA Lower cost per sample
Ion PGM^TM^	<400	30–2 Gb	<98%	Higher read quality Cost-effective
MinION	short to ultra-long (>4 Mb) reads	300–15 Gb	>95%	Resolving repetitive regions, G + C rich regions, and indels Portable
PacBio RS II (Single molecule real-time)	>20,000	1–10 Gb	>99.999%	Higher read quality Resolving repetitive regions, G + C rich regions, and indels

**Table 2:** Current and potential benefits of WGS in the management of NTM infections.

Accurate diagnostics of NTM infection within a clinically relevant time frame
Unrestricted classification of NTM subspecies compared to other genotyping methods
Characterization of the resistance profile and identification of novel gene mutation encoding resistance
Setting the appropriate combination of antibiotics and increasing the effectiveness of the treatment regimen
Monitoring of transmission dynamics and clustering of NTM in hospital settings to prevent nosocomial infections
Description of new genes affecting the pathogenesis of NTM infections
Development of new drugs
